# Supplementary material for: Phenolic Profile, Antioxidant Activity, and Chemometric Classification of Carob Pulp and Products
Source: Molecules. 2023 Feb 28;28(5):2269. doi: 10.3390/molecules28052269 (PMC10005499; doi:10.3390/molecules28052269)
Supplement: Supplementary file 1 [file molecules-28-02269-s001.zip › molecules-2227842-supplementary.pdf]

**Table S1:** Total phenolic content and antioxidant activity of carobs and carob-derived products.

| Assays | Carob pulp | | | Carob Powder | | | | Carob syrup | | |
|---|---|---|---|---|---|---|---|---|---|---|
| | Unroasted | | Roasted | Roasted | | | Unroasted | > 40 °C | | < 40 °C |
| | Cyprus | Greece | | Italy | Cyprus | Greece | | Cyprus | Greece | |
| TPC (mg GAE/ g product) | 7.20 ± 0.51 $^{i}$ | 7.69 ± 0.08 $^{i}$ | 12.28 ± 0.21 $^{f}$ | 23.18 ± 0.55 $^{a}$ | 21.04 ± 0.21 $^{c}$ | 21.82 ± 0.76 $^{b}$ | 18.19 ± 0.26 $^{d}$ | 11.29 ± 0.67 $^{g}$ | 9.51 ± 0.72 $^{h}$ | 14.79 ± 0.64 $^{e}$ |
| TPC (mg GAE/ g extract) | 14.92 ± 0.58 $^{fg}$ | 16.21 ± 0.39 $^{f}$ | 26.41 ± 0.14 $^{d}$ | 47.46 ± 0.74 $^{b}$ | 51.10 ± 0.68 $^{a}$ | 50.60 ± 0.71 $^{a}$ | 38.89 ± 2.47 $^{c}$ | 15.95 ± 0.72 $^{f}$ | 14.25 ± 0.69 $^{g}$ | 20.83 ± 0.60 $^{e}$ |
| DPPH ($IC_{50}$ mg extract/ mL) | 488.47 ± 34.67 $^{a}$ | 466.17 ± 18.13 $^{b}$ | 361.00 ± 11.92 $^{d}$ | 98.83 ± 3.20 $^{g}$ | 132.69 ± 1.04 $^{f}$ | 143.85 ± 1.97 $^{f}$ | 401.66 ± 1.06 $^{c}$ | 149.72 ± 9.40 $^{f}$ | 388.05 ± 1.75 $^{c}$ | 232.21 ± 19.58 $^{e}$ |
| FRAP (μmol TE/g product) | 73.94 ± 3.84 $^{de}$ | 71.49 ± 2.61 $^{e}$ | 89.22 ± 4.71 $^{c}$ | 144.32 ± 7.16 $^{a}$ | 138.24 ± 12.92 $^{a}$ | 140.84 ± 3.52 $^{a}$ | 119.41 ± 6.54 $^{b}$ | 48.58 ± 2.24 $^{f}$ | 46.36 ± 6.48 $^{f}$ | 82.23 ± 15.81 $^{cd}$ |

Different lower-case letters within the same row indicate significant differences ($p < 0.05$) according to Duncan's multiple range test.

**Table S2:** Data Matrix

| Sample | Sample Name | Matrix |
|---|---|---|
| S 1-3 | Carob pulp – Cyprus | Pulp |
| S 4-6 | Carob pulp – Greece | Pulp |
| S 7-9 | Roasted carob pulp – Greece | Pulp |
| S 10-12 | Roasted carob powder – Italy | Powder |
| S 13-15 | Roasted carob powder – Cyprus | Powder |
| S 16-18 | Roasted carob powder – Greece | Powder |
| S 19-21 | Unroasted carob powder – Greece | Powder |
| S 22-24 | Syrup thermally treated – Cyprus | Syrup |
| S 25-27 | Syrup thermally treated – Greece | Syrup |
| S 28-30 | Syrup non-thermally treated – Greece | Syrup |

**Table S3:** Correlation matrix of original variables (phenolics, TPC, antioxidant activity).

| | Folin | DPPH | FRAP | Gallic acid | catechin | epicatechin | caffeic acid | rutin | sinapic acid | quercitrin | myricetin | quercetin |
|---|---|---|---|---|---|---|---|---|---|---|---|---|
| Folin | 1 | -0,631072 | 0,951161 | -0,00117429 | 0,34368 | 0,481435 | -0,256159 | 0,419428 | 0,495681 | 0,0622677 | 0,0328528 | -0,0353748 |
| DPPH | | 1 | -0,447125 | -0,273648 | 0,148371 | -0,0317147 | 0,133833 | 0,2235 | -0,296638 | 0,274091 | 0,143161 | 0,144431 |
| FRAP | | | 1 | -0,153003 | 0,463212 | 0,598998 | -0,347974 | 0,611922 | 0,447205 | 0,128829 | 0,109979 | 0,121862 |
| Gallic acid | | | | 1 | -0,649402 | -0,272482 | 0,378349 | -0,536898 | -0,0518748 | -0,309805 | -0,207239 | -0,456781 |
| catechin | | | | | 1 | 0,68043 | -0,445349 | 0,849735 | 0,185749 | 0,704409 | 0,433702 | -0,100228 |
| epicatechin | | | | | | 1 | -0,217963 | 0,872591 | 0,421761 | 0,433816 | 0,31991 | -0,211283 |
| caffeic acid | | | | | | | 1 | -0,359962 | -0,10515 | -0,172415 | -0,114351 | -0,178144 |
| rutin | | | | | | | | 1 | 0,289021 | 0,495648 | 0,374112 | 0,0234648 |
| sinapic acid | | | | | | | | | 1 | -0,0300129 | 0,00140592 | -0,241328 |
| quercitrin | | | | | | | | | | 1 | 0,645592 | -0,265714 |
| myricetin | | | | | | | | | | | 1 | -0,137102 |
| quercetin | | | | | | | | | | | | 1 |

**Table S4:** Misclassification table of OPLS-DA.

| M2 | Members | Correct | pulp | powder | syrup | No class (YPred <= 0) |
|---|---|---|---|---|---|---|
| **pulp** | 9 | 100% | 9 | 0 | 0 | 0 |
| **powder** | 12 | 100% | 0 | 12 | 0 | 0 |
| **syrup** | 9 | 100% | 0 | 0 | 9 | 0 |
| **No class** | 0 | | 0 | 0 | 0 | 0 |
| Total | 30 | 100% | 9 | 12 | 9 | 0 |
| Fisher's prob. | 4,8e-13 | | | | | |

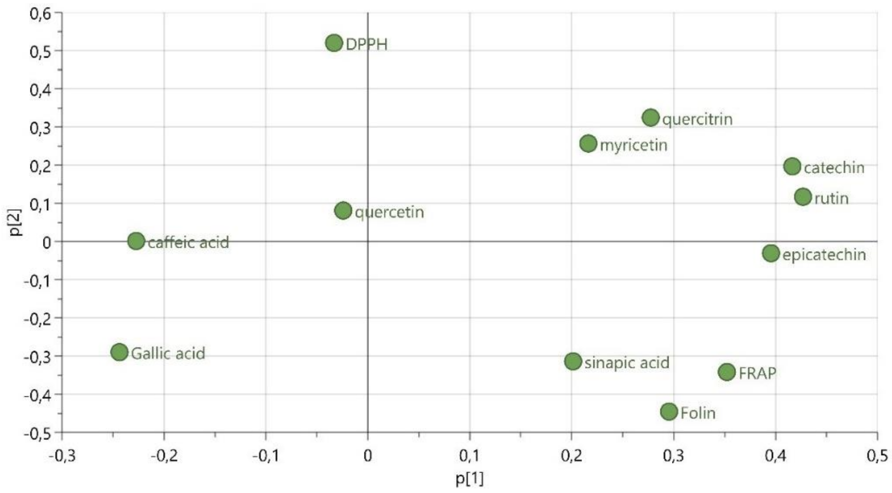


**Figure S1:** Loading score plot of the PCA model.

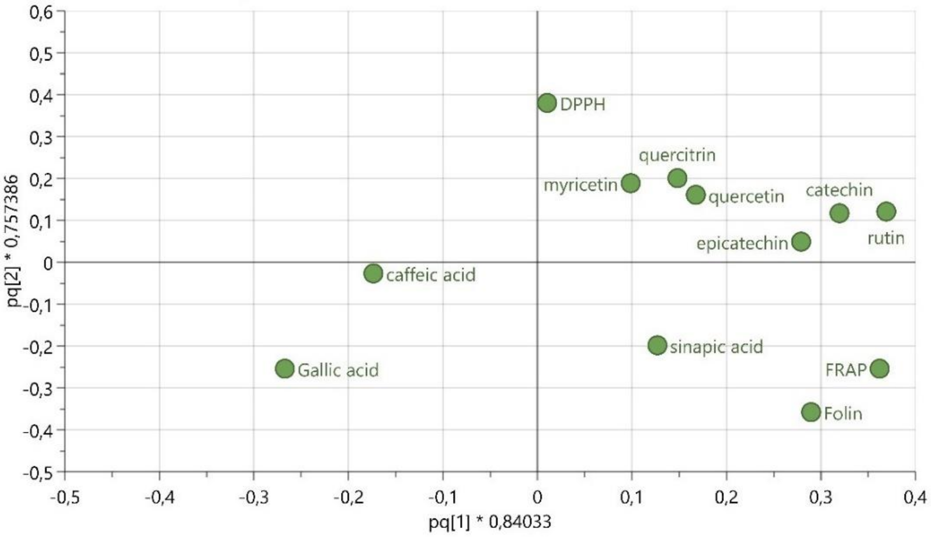


**Figure S2:** Loading score plot of the OPLS-DA model.
